# Discovery and characterization of a novel extremely acidic bacterial *N*-glycanase with combined advantages of PNGase F and A

**DOI:** 10.1042/BSR20140148

**Published:** 2014-11-14

**Authors:** Ting Wang, Zhi P. Cai, Xiao Q. Gu, Hong Y. Ma, Ya M. Du, Kun Huang, Josef Voglmeir, Li Liu

**Affiliations:** *Glycomics and Glycan Bioengineering Research Center, College of Food Science and Technology, Nanjing Agricultural University, 1 Weigang, Nanjing 210095, People's Republic of China; †Department of Plant Pathology, Nanjing Agricultural University, 1 Weigang, Nanjing 210095, People's Republic of China

**Keywords:** carbohydrate processing, core fucosylation, glycoprotein structure, *N*-linked glycosylation, PNGase, Terriglobus, 2-AB, 2-aminobenzamide, Asn, asparagine, CBB, Coomassie Brilliant Blue, HRP, horseradish peroxidase, MMXF3, Manα1-6(Manα1-3)(Xylβ1-2)Manα1-4GlcNAcα1-4(Fucα1-3)GlcNAc, MMX, Manα1-6(Manα1-3)(Xylβ1-2)Manα1-4GlcNAcα1-4GlcNAc, Ni-NTA, Ni^2+^-nitrilotriacetate, PMSF, phenyl-methylsulfonyl fluoride, PNGase, peptide *N*-glycosidase, UPLC, ultra-performance liquid chromatography

## Abstract

Peptide-N4-(*N*-acetyl-β-glucosaminyl) asparagine amidases [PNGases (peptide *N*-glycosidases), *N*-glycanases, EC 3.5.1.52] are essential tools in the release of *N*-glycans from glycoproteins. We hereby report the discovery and characterization of a novel bacterial *N*-glycanase from *Terriglobus roseus* with an extremely low pH optimum of 2.6, and annotated it therefore as PNGase H^+^. The gene of PNGase H^+^ was cloned and the recombinant protein was successfully expressed in *Escherichia coli.* The recombinant PNGase H^+^ could liberate high mannose-, hybrid- and complex-type *N*-glycans including core α1,3-fucosylated oligosaccharides from both glycoproteins and glycopeptides. In addition, PNGase H^+^ exhibited better release efficiency over *N*-glycans without core α1,3-fucose compared with PNGase A. The facile expression, non-glycosylated nature, unusual pH optimum and broad substrate specificity of this novel type of *N*-glycanase makes recombinant PNGase H^+^ a versatile tool in *N*-glycan analysis.

## Abstract 2

PNGase H^+^, from *T. roseus* was successfully cloned and expressed in *E. coli*. It shows activity in extremely acidic conditions and can release all types of *N*-glycans including core α1,3-fucosylated *N*-glycans from glycoproteins and glycopeptides in high efficiency.

## INTRODUCTION

**Figure 1:**
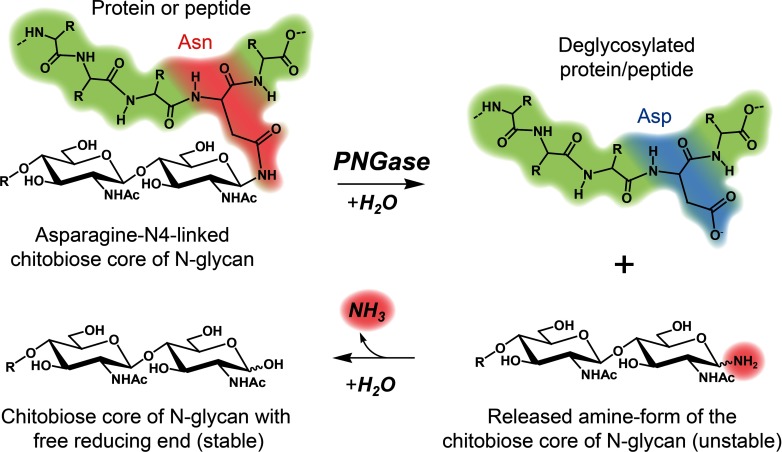
General reaction scheme for the enzymatic release of *N*-glycans by PNGases

*N*-glycanases are crucial tools in the structural and functional analyses of *N*-glycomes [1,2]. As shown in Figure 1, the catalytic function of this enzyme is the hydrolysis of a β-aspartylglucosaminyl bond between the core-chitobiose region of an *N*-glycan and an Asn (asparagine) residue, which results in the release of *N*-glycan moieties from glycoproteins or glycopeptides. Currently, only two different types of PNGases (peptide *N*-glycosidases) are commonly used in *N*-glycan analysis: PNGase F (UniProt number: P21163) from the bacterium *Flavobacterium meningosepticum* [3,4], and PNGase A (UniProt number: P81898) isolated from almonds (*Prunus dulcis*) [5].

Although the technique of using PNGase A and F in glycoprotein structure analysis has already become well established over the last three decades, some unsatisfied demands still remain. Recombinant PNGase F is expressed using *Escherichia coli* and is extensively used for deglycosylation of glycoproteins of mammalian origin. The activity of PNGase F, however, is hindered when core α1,3-fucose is present, a modification that is a typical feature of plant and invertebrate glycans [6,7]. In comparison to PNGase F, PNGase A has a broader substrate spectrum [8], allowing the release of *N*-linked oligosaccharides containing core α-1,3 fucose from glycopeptides [9,10]. However, PNGase A shows lower efficiency in *N*-glycan release from glycoproteins [11]. Furthermore, no heterologous expression of PNGase A in recombinant expression systems has yet been reported. So far, the only way to obtain PNGase A is extraction from almond seeds and consecutive purification using column chromatography. Moreover, PNGase A is a glycoprotein itself and therefore can be self-deglycosylated, which can bias the outcome of the *N*-glycan analysis by causing contamination from endogenous PNGase A glycan structures [12]. In comparison, PNGase F can be cost-effectively produced on a large scale in prokaryotic hosts in an insoluble form, which yields after protein refolding an unglycosylated active enzyme of high purity that is even suitable for proteomics studies [13]. Considering the rapid development of *N*-glycoproteome studies, especially in plants, there is an urgent need for novel types of PNGases that combine the advantages of both PNGase A and PNGase F [14]. To find a PNGase candidate with a broad substrate spectrum like PNGase A, which can release all types of *N*-glycans including those of plant and invertebrate species, and which can also be conveniently expressed in *E. coli*, like PNGase F, is a major challenge.

Several homologues of PNGase A have been discovered in plants and fungi [10,15]. For example, Plummer's group discovered a PNGase from *Aspergillus tubigensis* (PNGase At, GenBank accession number: U96923.1) [15]. PNGase At is a homologue of PNGase A that also possesses a similar substrate spectrum. Nevertheless, recombinant PNGase At is also a glycoprotein and is only successfully expressed in baculovirus-infected insect cell systems. The Kimura group identified another candidate from tomato fruits, PNGase Le (UniProt number: D0QU16), which is an acidic PNGase. This enzyme could be heterologously expressed in active form in *Pichia pastoris*; however, expression of active PNGase Le in *E. coli* failed [16].

Since the start of this millennium, the genomic information of thousands of micro-organisms has been revealed [17]. Mining these vast amounts of genomic data for sequence homologues with known PNGase isoforms is an applicable approach to find novel PNGase candidate genes of bacterial origin. This approach helped us to discover several putative PNGase genes in various strains from the newly devised phylum *Acidobacteria* during screening for PNGase A homologues in bacteria from GenBank [18]. In this report, we describe the gene mining, cloning, expression and characterization of a novel PNGase from *Terriglobus roseus* DSM 18391 (PNGase H^+^). The recombinant enzyme shows high enzymatic activities at extremely low pH values, and accepts a broad range of both glycoprotein and glycopeptide substrates.

## EXPERIMENTAL

### Materials

*T. roseus* (strain DSM 18391) was obtained from the DSMZ (German Collection of Microorganisms and Cell Cultures). Oligonucleotide primers were synthesized by GenScript Co. Ltd. The dabsylated glycopeptide, dabsyl–Gly–Glu–Asn–(GlcNAc_4_Man_3_)–Arg was kindly provided by Mr Thomas Dalik and Dr Friedrich Altmann (University of Natural Resources and Applied Life Sciences, Vienna, Austria). All the reagents and chemicals used were of the highest grade available from the suppliers.

### Amplification of PNGase H^+^ gene and construction of the expression plasmid

Genomic DNA was isolated from the lyophilized culture sample of *T. roseus* DSM 18391 according to the method described by Mahuku et al. [19]. The primers were designed based on the genomic DNA sequence of *T. roseus* DSM 18391 (GenBank Accession Number: CP003379.1), primer PNGase H^+^ Forward: 5′-atacatatgCCCCGCATCTTGTGCCGCCCT-3′ (sense primer with the Nde I restriction site) and primer PNGase H^+^ Reverse: 5′-atactcgagGCGTTTCACCGGGCAGCCTGC-3′ (antisense primer with the Xho I restriction site). The gene encoding PNGase H^+^ was amplified by PCR using PrimerSTAR™ (Takara) DNA polymerase according to the manufacturer's instructions. Briefly, the 30 PCR cycles consisted of denaturation at 95°C for 10 s, annealing at 70°C for 30 s and extension at 72°C for 2 min. The PCR products were digested with Nde I and Xho I restriction endonucleases (Thermo Scientific) and ligated into a pET30a expression vector (Novagen), which was also digested with the same two restriction enzymes. The recombinant vector pET30a/PNGase H^+^ was transformed into *E. coli* Top10 competent cells (Invitrogen) and plated onto LB agarose plates supplemented with kanamycin as selection marker. Plasmids isolated from clones which contained the expected sequence after sequencing confirmation of the insert were used in further experiments. The extraction of plasmids, digestion with restriction enzymes, ligation and transformation were carried out using the standard methods unless mentioned otherwise.

### Expression and purification of PNGase H^+^

Plasmids bearing the expected gene sequence were transformed into *E. coli* BL21(DE3) competent cells (Invitrogen) and selected transformants were grown in 1000 ml LB media at 37°C in an incubator shaker at 250 rpm until the culture density reached an OD_600_ value of 0.8. Recombinant protein expression was induced by adding IPTG (isopropyl β-D-thiogalactopyranoside) to the final concentrations of 0.1, 0.5 or 1.0 mM. Induction was carried out at 18 or 25°C for 3, 9, 15 and 24 h before harvest. The cell pellets (approximately 1.5 g of wet weight) were collected by centrifuging at 5000 ***g*** for 15 min, resuspended in 10 ml cell lysis solution containing 5% acetic acid (v/v), 1% Triton X-100 (v/v) and 1 mM PMSF (phenyl-methylsulfonyl fluoride) and then sonicated for 20 min (40 on/off cycles with 20 μm amplitude for 15 s at 4°C). The cell lysate was centrifuged for 30 min at 20 000 ***g*** and the supernatant was collected. Before loading onto the Ni-NTA (Ni^2+^-nitrilotriacetate) agarose, the pH value of crude extract was adjusted to 7.0 with 1 M NaOH solution. The nickel-affinity purification was performed according to the manufacturer's instructions (Qiagen). Sample aliquots were further incubated with 2×Laemmli buffer at 95°C for 10 min, separated by SDS/PAGE and visualized by CBB (Coomassie Brilliant Blue) G-250 staining. The concentration of the purified recombinant protein was determined using a Bradford Protein Assay Kit according to the manufacturer's instructions (Sangon Biotech).

### UPLC (ultra-performance liquid chromatography)-based activity assay of PNGase H^+^

A dabsylated glycopeptide purified from fetuin [dabsyl–Gly–Glu–Asn–(GlcNAc_4_Man_3_)–Arg] was used as a substrate to test the activity of PNGase H^+^. Typically, a reaction mixture of 75 μl contained 0.5 μl of purified PNGase H^+^ enzyme solution, 3 μl of glycopeptide substrate (10 pmol/μl), 37.5 μl of glycine/HCl buffer (0.4 M, pH 2.6) and 34 μl of distilled water. An assay mixture with water instead of the PNGase H^+^ enzyme solution was used as a negative control. PNGase F (Promega) treatment was used as a positive control, which contained 2 μl PNGase F (1 mU, which is defined by the manufacturer as the amount of enzyme required to catalyse the release of *N*-glycans from 1 μmol of denatured RNAse B per minute at pH 7.5 and 37°C) enzyme solution, 3 μl of glycopeptide substrate (10 pmol/μl), 7.5 μl 500 mM phosphate buffer, pH 7.5, 1.5 μl 10% Triton X-100 (v/v) and 62.5 μl distilled water. The reactions were incubated at 37°C for 10 min, 30 min, 1 h, 4 h and overnight, and then quenched by heating at 95°C for 5 min. Chromatographic analyses were performed on a Shimadzu Nexera UPLC system (Shimadzu Corporation) consisting of an LC-30AD pump equipped with a low-pressure gradient-mixing unit, an SIL-30AC autosampler and an SPD-UV20AD absorbance detector. The analytes were separated on a reversed-phase UPLC column (Phenomenex Kinetex® 1.7 μm C18 100 Å 150×2.10 mm^2^) at a constant flow rate of 0.5 ml/min and analysed by UV/Vis detection at 436 nm. Solvent A was 0.1% (v/v) formic acid in water and solvent B was 0.1% (v/v) formic acid in acetonitrile. After injection of 30 μl of sample, a linear gradient of 5–40% B was applied from 0 to 5 min, then B was increased to 77% over 10 min and held at 77% for 1 min. B was then decreased to 5% in 1 min and the column was equilibrated with the initial conditions (5% of B) for 5 min.

### Characterization of the recombinant PNGase H^+^

The optimum pH value of PNGase H^+^ was determined by incubating PNGase H^+^ with dabsyl–Gly–Glu–Asn–(GlcNAc_4_Man_3_)–Arg in different buffers with various pH values at 37°C for 1 h according to the activity assay described above. The buffers used were glycine/HCl (0.2 M final concentration, final pH 2.0, 2.6 or 3.0), citrate/NaOH (50 mM final concentration, final pH 3.5, 4.0, 4.5, 5.0 or 5.5) and MES buffer (50 mM final concentration, final pH 6.0, 6.5 and 7.0). The activity of the recombinant PNGase H^+^ was also tested in an assay mixture where the buffer was replaced with acids to obtain the reaction mixtures containing either 500 mM acetic acid, 500 mM formic acid or various concentrations of citric acid and hydrochloric acid (500 or 100 mM final concentration). The optimum temperature of the recombinant PNGase H^+^ was determined by incubation of the standard reaction mixture at different temperatures ranging from 4 to 80°C in 0.2 M glycine/HCl buffer (pH 2.6). Different metal salts (CoCl_2_, CaCl_2_, FeCl_2_, MgCl_2_, CuCl_2_ and ZnCl_2_) and EDTA were added at a final concentration of 10 mM to the reaction mixture to study the effects of metal ions on the activity of the recombinant PNGase H^+^. The effect of different denaturants and detergents on the enzymatic activity, namely 2-mercaptoethanol (10, 50 or 100 mM final concentration), Triton X-100 (0.1, 0.5 and 1%, v/v) and urea (0.5, 1 or 2 M final concentration), were also estimated using this method. The reactions were analysed by the UPLC-based activity assay as described above, and the activity was determined by conversion rates relative to standard condition assay mixtures (glycine/HCl buffer, pH 2.6).

### Gel-based deglycosylation assay

The glycoproteins were denatured by heating at 95°C for 10 min in distilled water. A 40 μl reaction mixture contained 1 μl of recombinant PNGase H^+^ (1.2 μU), 10 μl of denatured glycoprotein (1 μg/μl), 20 μl of glycine/HCl buffer (0.4 M, pH 2.6) and 19 μl of distilled water. The positive control contained 1 μl PNGase F (2 mU), 10 μl of denatured glycoprotein (1 μg/μl), 4 μl sodium phosphate buffer (0.5 M, pH 7.5) buffer and 35 μl distilled water. The assay mixture was incubated at 37°C overnight and the reaction was terminated by the addition of 10 μl of 2×Laemmli buffer and subsequently heating at 95°C for 10 min. The samples were visualized by CBB-stained SDS/PAGE using the same conditions as described above.

### UPLC-based *N*-glycan profiling

HRP (horseradish peroxidase), RNase B and lactoferrin were chosen as model glycoproteins in this study to cover all types of *N*-glycans including high-mannose, hybrid and complex types of *N*-glycans. For glycopeptide experiments, glycoproteins (typically 100 μg in water) were first heated at 95°C for 10 min. Subsequently, the glycoproteins were cooled down and proteolytically digested with either pepsin (in 5% formic acid, v/v) or trypsin (in 200 mM ammonium bicarbonate buffer, pH 8.0) for 16 h at 37°C with a protease:glycoprotein ratio of 1:25 (w/w). The reactions were stopped by heating at 95°C for 10 min and dried by vacuum concentration. The glycopeptide samples were then incubated with PNGase H^+^ (12 μU) in 0.2 M glycine/HCl buffer pH 2.6 at 37°C overnight. PNGase F was used as the positive control for RNase B and lactoferrin deglycosylation treatments according to the manufacturer's instruction (0.1% SDS (w/v), 50 mM 2-mercaptoethanol and 10 μl PNGase F (20 mU), 1% Triton X-100 (v/v) and 50 mM sodium phosphate buffer (pH 7.5) at 37°C for 16 h). PNGase A (Prozyme) was used as the positive control for the HRP deglycosylation reaction according to the supplier's instructions (0.5 mU, one unit is defined as the amount of enzyme required to release 1 μmol ovalbumin per min at pH 5.0 and 37°C), while PNGase F was used as the negative control in this case.

For glycoprotein experiments, 100 μg of glycoproteins were first heated at 95°C for 10 min. PNGase H^+^ was then added to all three samples and the reactions were performed under the same condition as described above. Again, PNGase F was used as the positive control for RNase B and lactoferrin experiments. PNGase A was used as the positive control and PNGase F was used as the negative control for HRP deglycosylation treatment. For all PNGase F treatments, the samples were alternatively denatured by preheating with 0.1% SDS (w/v) and 50 mM 2-mercaptoethanol for 10 min prior to the enzymatic digestion according to the supplier's instruction. All reactions were stopped by heating at 95°C for 10 min and dried by vacuum concentration. The dry samples were fluorescently labelled with 2-AB (2-aminobenzamide) using the SIGNAL™ 2-AB labelling kit (Prozyme) according to the manufacturer's instructions. 2-AB labelled glycans were analysed by normal-phase UPLC (Shimadzu Nexera) equipped with the same parts as described previously except for the alternative utilization of a fluorescence detector (RF-20Axs) and an Acquity BEH Glycan column (Waters, 1.7 μm, 2.1×150 mm). The effluent was monitored by the fluorescence detector (Ex=330 nm, Em=420 nm) and the flow rate was 0.5 ml/min. Solvent A was aqueous 50 mM ammonium formate buffer, pH 4.5 and solvent B was acetonitrile. A linear gradient of 95–78% of B was applied from 0 to 6 min, B was then decreased to 56% over 39 min followed by further decrease to 0% over 3 min and held at 0% for 2 min. B was then increased to 95% in 2 min and the column was equilibrated with the initial conditions for 7 min. UPLC fractions were manually collected and the selected peaks were subjected to MS analysis.

### MS glycan analysis

Monoisotopic MALDI–TOF-MS (matrix-assisted laser-desorption ionization–time-of-flight MS) spectra were acquired using a Bruker Ultraflex Extreme TOF–TOF instrument with 6-aza-2-thiothymine as matrix. Mass Spectra were processed using Bruker Flexanalysis version 3.3 and were analysed manually, and the annotation of mass peaks from MS and MS–MS were further evaluated using GlycoWorkbench version 1.1 [20].

## RESULTS

### Gene cloning of the PNGase H^+^ gene and construction of expression plasmid

The full-length ORF (open reading frame) encoding of the PNGase H^+^ gene could be successfully cloned and consists of 1707 bps (Supplementary Figure S1). It encodes a putative protein of 570 amino acids with a theoretical molecular mass of 60.7 kDa. The recombinant PNGase H^+^ contains an additional hexa-histidine tag at the C-terminal end to facilitate the protein purification.

### Expression and purification of the recombinant PNGase H^+^

**Figure 2:**
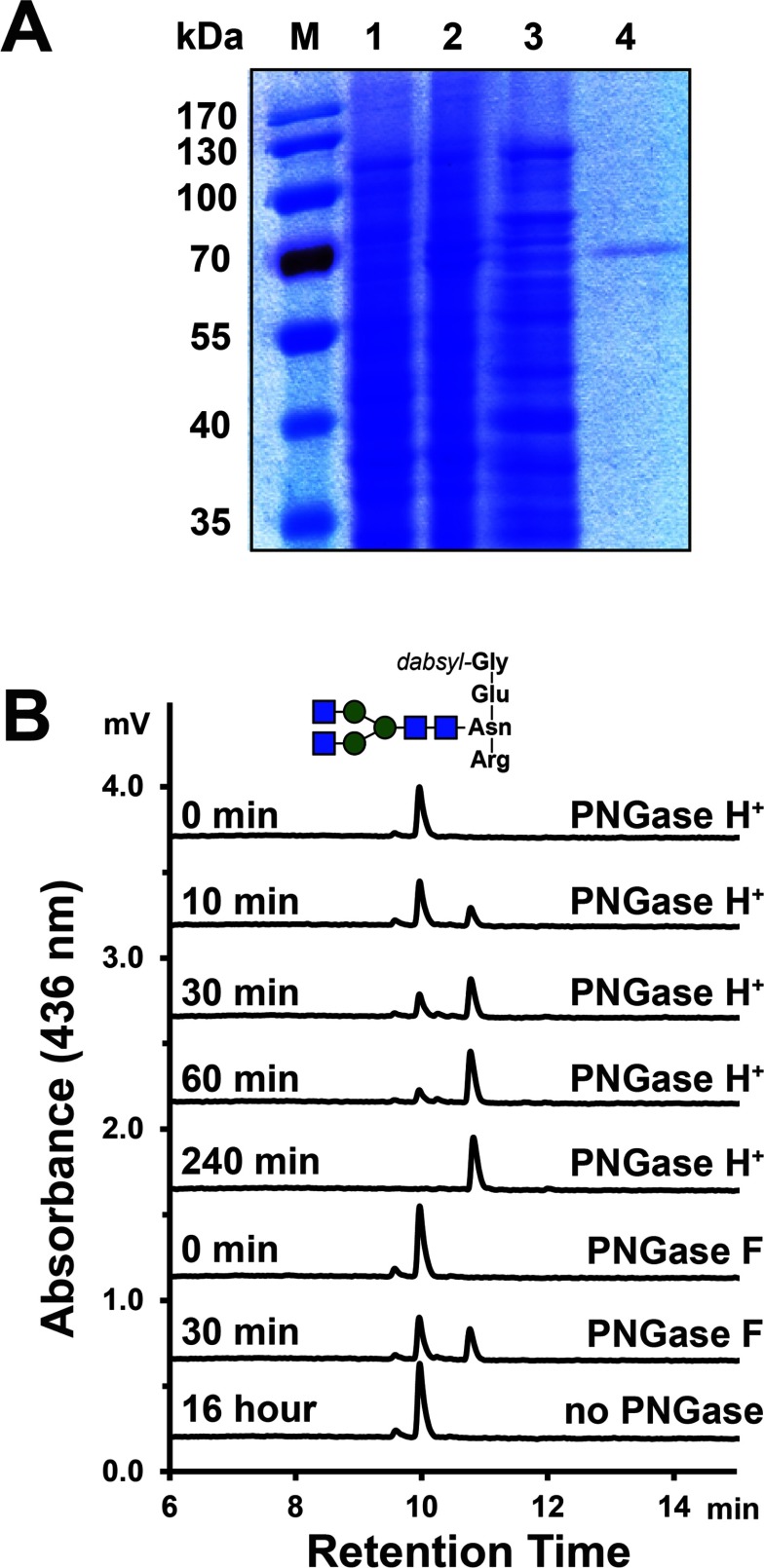
SDS/PAGE and the glycopeptide assay of recombinant PNGase H^+^ (**A**) SDS/PAGE analysis of heterologous expression of recombinant PNGase H^+^ in *E. coli*. Lane M, protein marker; Lane 1, cell pellet before inducing; Lane 2, cell pellet after inducing with IPTG; Lane 3, acidic supernatant after cell lysis; Lane 4, Ni-NTA purified recombinant PNGase H^+^. (**B**) Chromatography of the glycopeptide based activity.

The best expression results for recombinant PNGase H^+^ were obtained by inducing cells for 24 h at 18°C with 1 mM IPTG. Enzymatic activity could be already detected from the crude *E. coli* cell lysates. However, the enzyme was rapidly degraded after cell lysis in Tris/HCl buffer which is commonly used in cell disruption procedures, although a protease inhibitor was already added. Replacing the buffer with 5% of acetic acid solution (containing 1% Triton X-100 and 1 mM PMSF prevented the degradation of the recombinant PNGase H^+^. Fractions from the nickel-affinity purification of the re-neutralized lysis supernatant were tested for enzymatic activity. The active fractions showed a single band with molecular mass over 70 kDa on SDS/PAGE (Figure 2A) and were used for further enzymatic characterization. The purified enzyme retained almost full activity after 2 months of storage in the elution buffer (500 mM NaCl in 50 mM Tris/HCl buffer, pH 8.0) at 4°C. The concentration of the purified enzyme was 26 μg/ml.

### Characterization of PNGase H^+^

The enzymatic properties of the recombinant PNGase H^+^ were determined using dabsyl–Gly–Glu–Asn–(GlcNAc_4_Man_3_)–Arg as the substrate, and the hydrolysis activity was monitored using the UPLC-based assay (Figure 2B). After 1 h of incubation with PNGase F (the positive control), the peak at 10 min retention time decreased and a second peak at 10.8 min appeared, which was considered to be the peptide part of the reaction product, dabsyl–Gly–Glu–Asp–Arg. A peak at the same retention time was observed for different time point incubations of the PNGase H^+^ reaction. The product peak continuously increased over time until the complete disappearance of the starting glycopeptide after 4 h incubation time, which indicated that all of the substrate had been converted to the product by PNGase H^+^. The activity of PNGase H^+^ determined by this assay was 1.2 mU/ml [1 unit was defined as the deglycosylation of 1 μmol of dabsyl–Gly–Glu–Asn–(GlcNAc_4_Man_3_)–Arg per minute at 37°C in 0.2 M glycine/HCl buffer, pH 2.6], and the activity of commercial PNGase F was 0.4 mU/ml [1 unit was defined as the deglycosylation of 1 μmol of dabsyl–Gly–Glu–Asn–(GlcNAc_4_Man_3_)–Arg per minute at pH 7.5 and 37°C]

**Figure 3:**
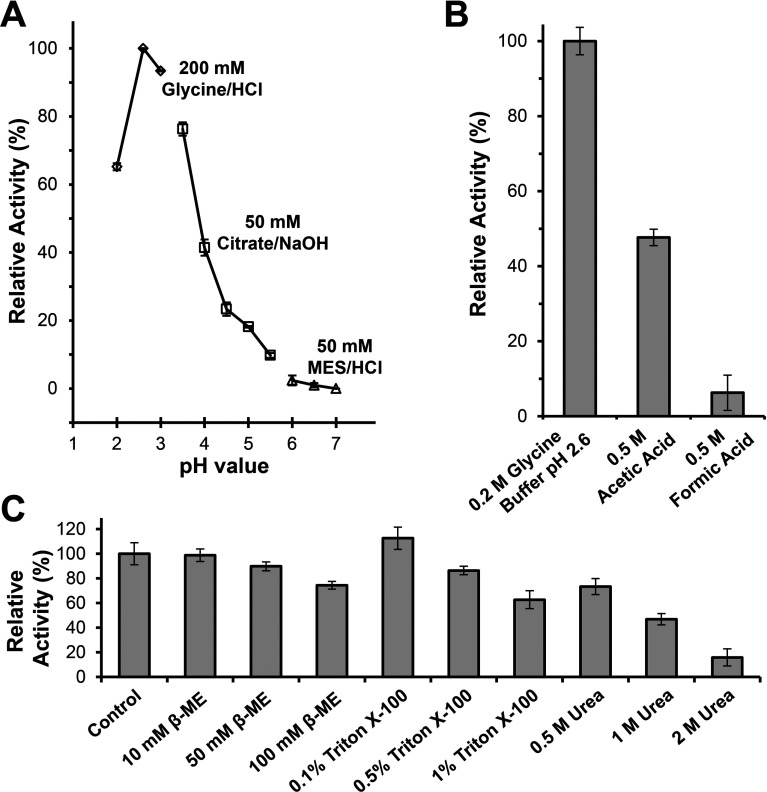
Effects of pH, organic acids and denaturants on the activity of recombinant PNGase H^+^ (**A**) Effects of pH on the activity of recombinant PNGase H^+^. (**B**) Effects of acids on the activity of recombinant PNGase H^+^. The relative activities of recombinant PNGase H^+^ in 0.5 M acetic acid and 0.5 M formic acid were calculated using 0.2 M glycine/HCl buffer, pH 2.6 as control. (**C**) Effects of denaturants on the activity of recombinant PNGase H^+^. The reaction in 0.2 M glycine/HCl buffer, pH 2.6 without any unfolding reagents was used as control.

The effects of pH, temperature, metal ions and detergents on the enzymatic activity of PNGase H^+^ were also investigated. Initial activity tests were performed at a pH value of 6.5 in MES buffer, in which the enzyme only displays moderate enzymatic activities. To our surprise, the pH optimization tests showed increasing conversion rates of the substrate with decreasing pH values, and the maximum activity of PNGase H^+^ was obtained at pH 2.6. As shown in Figure 3(A), there was a remarkable loss of activity above pH 3.5. To reconfirm the activity of the recombinant PNGase H^+^ in an acidic environment, enzymatic assays were also performed in reaction mixtures containing various acids at high concentrations. The results show that recombinant PNGase H^+^ in 500 mM acetic acid or 500 mM formic acid exhibited relative activities of 48 and 7.5%, respectively (Figure 3B), whereas no activity could be observed in 500 mM citric acid. However, repeating this assay with 100 mM citric acid, a relative conversion rate of 10% was observed. Furthermore, no enzymatic activity was observed in assay mixtures containing 100 mM hydrochloric acid.

The highest activity of recombinant PNGase H^+^ was achieved by incubating the assay mixtures at a 22°C. Slightly lower activities were observed at 30°C (86%) and 37°C (81%). The activity of the recombinant PNGase H^+^ decreased rapidly above 37°C. At 45, 55 and 70°C, the relative activities were determined to be 45, 1.1 and 0% of the activity at 22°C, respectively. The addition of various metal ions and EDTA showed no significant effects on activity (results not shown). The effect of 2-mercaptoethanol, Triton X-100 or urea on the activity of PNGase H^+^ was also tested (Figure 3C). Compared with the control (without any denaturants), the relative activities of PNGase H^+^ in 10, 50 and 100 mM 2-mercaptoethanol remained at 98, 90 and 74%, respectively. The relative activities in 0.5, 1 and 2 M urea were 73, 47 and 16%, respectively. Briefly 0.5 and 1% Triton X-100 decreased the activity of the recombinant PNGase H^+^ to 86 and 63%, respectively, while 0.1% Triton X-100 could increase the relative activity to 113%.

### Substrate specificity of PNGase H^+^

**Figure 4:**
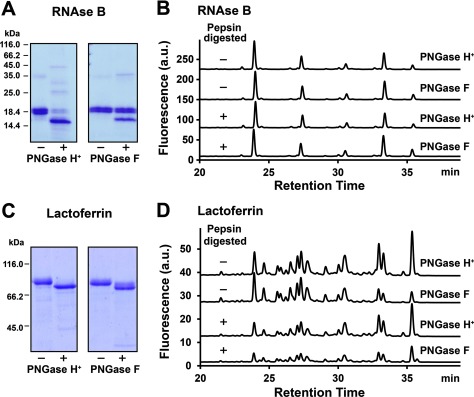
Substrate specificity tests of recombinant PNGase H^+^ using RNase B and lactoferrin (**A**) SDS/PAGE analysis of heat denatured RNase B with (+) and without (−) PNGase H^+^ and F. (**B**) UPLC chromatographs of *N*-glycans released from RNase B. PNGase F was chosen as control. Heat denatured RNAse B and pepsin digested RNase B were used as substrates. (**C**) SDS/PAGE analysis of heat denatured lactoferrin with (+) and without (−) PNGase H^+^ and F. (**D**) UPLC chromatographs of *N*-glycans released from lactoferrin. PNGase F was chosen as control. Heat denatured lactoferrin and pepsin digested lactoferrin were used as the substrates.

HRP, RNase B and lactoferrin were used for the substrate specificity test of the recombinant PNGase H^+^. SDS/PAGE was applied to monitor the deglycosylation capabilities of the recombinant PNGase H^+^ and PNGase F on heat-denatured glycoproteins without the addition of any denaturants. Assay mixtures without enzymes were used as negative controls. Heat-denatured RNase B was only partly deglycosylated by PNGase F, while it was almost completely deglycosylated by PNGase H^+^, as evidenced by the migration of the primary protein band from approximately 19 to 16 kDa (Figure 4A). In a similar manner, overnight incubations of heat-denatured lactoferrin with either PNGase F or PNGase H^+^ resulted in a complete shift of the primary protein band on the SDS/PAGE to a lower molecular mass compared with the negative control (Figure 4C).

UPLC profiles of the released *N*-glycans from denatured RNase B, denatured lactoferrin and *N*-glycans released from their glycopeptides derived by pepsin digestion are shown in Figures 4(B) and 4(D). HPLC profiles of the released *N*-glycans by PNGase H^+^ treatment from all substrates were shown to be similar to the ones obtained by PNGase F treatment. Moreover, the HPLC profiles obtained in this study are also shown to be comparable with that reported for PNGase F hydrolysis of the same glycoproteins in the literatures [21,22]. These results indicated that high-mannose-type, complex-type and hybrid-type *N*-glycans of mammalian origin can all be released with PNGase H^+^ in a similar manner as with PNGase F.

**Figure 5:**
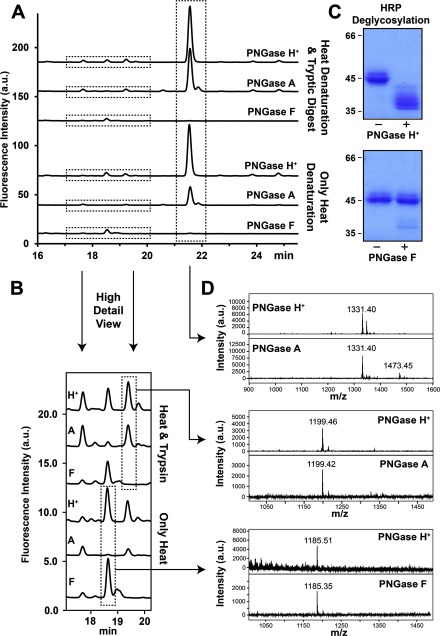
HRP N-glycan profiles using recombinant PNGase H^+^, A and F (**A**) UPLC chromatographs of *N*-glycans released from HRP. PNGase A and F were chosen as control. Heat denatured HRP and trypsin digested HRP were used as substrates. (**B**) Enlarged chromatographs of *N*-glycans released from HRP. (**C**) SDS/PAGE analysis of heat denatured HRP with (+) and without (−) PNGase H^+^ and F. (**D**) MALDI–TOF-MS analysis of selected *N*-glycan fractions derived from HRP.

To profile *N*-glycans of heat-denatured HRP glycoprotein samples and *N*-glycans of trypsin-derived HRP glycopeptide samples, comparative deglycosylation experiments were performed not only with PNGase F but also with PNGase A, as HRP bears the typical plant type *N*-glycans with the core α1,3-linked fucose motif. Remarkable differences were found among the glycan profiles of HRP treated by PNGase A, PNGase H^+^ and PNGase F (Figure 5A). The glycan profile from PNGase A and H^+^ treatments consisted of one main peak (elution time 21.4 min) which accounted for more than 80% of the released *N*-glycans based on calculated relative peak areas. MS analysis revealed that the *m*/*z* value of this main peak was 1331.40 Da [M+Na]^+^ (Figure 5D, upper part) which corresponds well to 2AB-Hex_3_HexNAc_2_Xyl_1_Fuc_1_, with a calculated monoisotopic mass of 1331.49 Da [M+Na]^+^. Further MS–MS fragmentation confirmed the core fucosylation of this structure and the linkage of xylose to the trimannoside portion of the glycan (Supplementary Figure S2A). This observation is in agreement with previous report that identified this glycan as the main *N*-glycan structure on HRP [23]. Noteworthy, a second smaller peak, immediately eluting after the main peak, with a retention time of 21.7 min was only observed in PNGase A treated HRP samples. It gave an *m*/*z* value of 1473.45 Da [M+Na]^+^ in the mass spectrum and does not correspond to any described HRP glycan structure so far. However, further MS–MS fragmentation indicated the *N*-glycosidic nature of this compound (Supplementary Figure S2B). Another difference between HRP samples treated with PNGase H^+^, A and PNGase F was observed in the chromatograms at the retention time 19.5 min, while no peak was observed at the *N*-glycans profile treated with PNGase F; both PNGase H^+^ and A showed a small minor peak. The *m*/*z* value of this peak was 1199.46 Da [M+Na]^+^ (Figure 5D, middle part) which corresponds to 2AB-Hex_3_HexNAc_2_Fuc, with a calculated monoisotopic mass of 1199.44 Da [M+Na]^+^. Further MS–MS fragmentation confirmed the core fucosylation of this structure (Supplementary Figure S2C). As expected, the glycan profile of PNGase F-treated HRP samples only showed minor peaks that contain no core α1,3-fucose. Higher release efficiencies for a peak at the retention time 18.5 min was observed in HRP samples treated with PNGase H^+^ and PNGase F in comparison with PNGase A (Figure 5B). MS analysis of this minor peak fraction revealed that the *m*/*z* value of this peak was 1185.4±0.1 Da [M+Na]^+^ (Figure 5D lower part), which corresponds to 2AB-Hex_3_HexNAc_2_Xyl_1_ (theoretical monoisotopic, sodiated mass 1185.43 Da) and was further confirmed by MS–MS fragmentation (Supplementary Figure S2D). This stands in agreement with reported data, which showed that PNGase F can also release core-xylosylated *N*-glycans without core α1,3-linked fucose [8]. Furthermore, a minor HRP glycan fraction having different release efficiencies for PNGase H^+^, A and F was observed in the chromatograms at the retention time 17.5 min. MS analysis of this purified fraction gave a weak *m*/*z* peak was 1023.41 Da [M+Na]^+^, indicating 2AB-Hex_2_HexNAc_2_Xyl as corresponding structure with a calculated monoisotopic mass of 1023.38 Da [M+Na]^+^ (results not shown). However, owing to the low signal response of this fraction no MS–MS fragmentation could be performed. In addition, deglycosylation experiments of heat-denatured HRP samples incubated with PNGase H^+^ and PNGase F were analysed by SDS/PAGE to monitor the deglycosylation capabilities of the enzymes. As shown in Figure 5(C), the protein band of 45 kDa in heat-denatured HRP completely shifted to several smaller discrete protein bands with masses between 35 and 40 kDa after PNGase H^+^ treatment, whereas only a negligible shift of the main protein band was observed after PNGase F treatment.

## DISCUSSION

The discovery of novel PNGases is an important step to further improve the analysis of the *N*-glycome. Considering the urgent demands in finding more suitable PNGase candidates, we compared PNGase A from almonds (Swissprot Accession Number P81898) by homology search of translated nucleotide databases (TBLASTN) of bacterial genomes. After careful consideration, a putative PNGase gene candidate from the soil bacteria *T. roseus* DSM 18391, encoding a protein that showed 28% sequence identity in comparison with PNGase A, was selected for further investigation. Initial purification trials of the recombinant PNGase H^+^ using Tris/HCl (pH 8.0) as buffer yielded only small amounts of purified protein after nickel affinity chromatography. Although enzymatic activity could be detected in these purified fractions, visualization by CBB-stained SDS/PAGE displayed several smaller protein bands, which are probably an indication of the proteolytic degradation of PNGase H^+^, although protease inhibitor was used. Several trials in further purifying the recombinant enzyme using size-exclusion chromatography (Sephadex G-75) or anion exchange chromatography (DEAE–Sepharose) were not successful. Despite the lack of protein purity, we decided to further investigate the optimization of our activity assay, which led us to try more acidic conditions for the purification protocol. As the enzymatic activity was higher in acidic conditions, we presumed that PNGase H^+^ might also have a certain tolerance towards acids, whereas proteases from the recombinant host should be less active in this environment. The subsequent neutralizing of the pH of the supernatant before loading it onto the nickel-affinity column was a necessary step to maintain the optimal binding capacity of the resin. The recombinant PNGase H^+^ could be successfully purified using this method and was observed as a single protein band on SDS/PAGE. However, the calculated molecular mass of 61.5 kDa did not correspond exactly with the observed size which was slightly below the 70 kDa protein band of the marker. The same size was observed by anti-His-tag Western blot analysis of whole-cell protein from induced cell pellets (results not shown).

A possible explanation for the activity of this enzyme in acidic conditions is that *T. roseus* might secrete this enzyme into an acidic extracellular environment. A secondary structure analysis using the SignalP sequence prediction server (http://www.cbs.dtu.dk/services/SignalP) [24] indicated that the *N*-terminus of PNGase H^+^ contains a 28 amino acid long secretion signal. As the role of *Terriglobus* species in soil ecosystems is not clearly understood yet, one can only speculate if the action of PNGase H^+^ fulfils the nutritional, defensive, pathogenic or symbiotic purposes for the host. *Terriglobus* strains can, in contrast to other isolates from the phylum *Acidobacteria*, also grow under mildly acidic conditions [25]. Most acidic PNGases were described to have a pH optimum between 4.0 and 5.0 [15,16,26,27], which is probably based on the slightly acidic environment in vacuoles or their extracellular environment after secretion [15,28].

Cytoplasmic PNGases are described to be dependent on divalent zinc ions for full enzymatic activity [29]. In contrast, PNGase H^+^ showed similar properties to PNGase A and PNGase F [30,31], which also show no metal ion requirements. The decreased activity of PNGase H^+^ above 37°C could be explained in a similar manner as the pH optimum is based on the environmental conditions which soil bacteria are exposed to: the optimum growth temperature for *T. roseus* was reported to be 25°C, with growth fully inhibited at 37°C [25].

The *N*-glycoprofiles of glycoproteins treated with PNGase H^+^ showed a highly similar pattern to the *N*-glycoprofiles derived by PNGase F treatment for RNase B and lactoferrin. RNase B was reported to contain various isoforms of the high mannose glycoforms including Man5–Man9 [32] and was therefore a good representation for deglycosylation experiments of this type of *N*-glycans. The obtained *N*-glycoprofile in this study stands also in good agreement with reported data for RNase B glycoprofiles by others [21]. PNGase H^+^ was therefore shown to have the similar capability as PNGase F when releasing high-mannose type of *N*-glycans from glycoproteins and glycopeptides. As the *N*-glycans of lactoferrin reportedly contain approximately 35% complex- and hybrid-type structures [22], this glycoprotein was chosen to further investigate the substrate specificity of PNGase H^+^. Obtained *N*-glycoprofiles in this study showed the same HPLC patterns of *N*-glycans between PNGase H^+^ and PNGase F treatments. In addition, the results also exhibited a significant correlation in comparison with reported normal-phase HPLC glycoprofiles released by PNGase F [22]. It can thus be concluded that PNGase H^+^ is a good alternative to PNGase F for the liberation of different types of *N*-glycans without core α1,3-linked fucose.

Considering the acidic condition of the enzymatic *N*-glycan release using PNGase H^+^, acid-sensitive moieties such as terminal sialic acids might be effected. In order to prove that sialic acids are still present after overnight incubation in glycine/HCl buffer, PNGase H^+^ treatment of the sialylated glycoprotein fetuin was performed. UPLC analysis of this *N*-glycans released from fetuin confirmed the presence of sialylated *N*-glycans by comparison with sialidase-treated *N*-glycans from fetuin (results not shown). However, as the partial removal of sialic acids at these low pH values cannot be entirely avoided, further method optimization using PNGase H^+^ will be required to find a suitable balance between maintaining the enzymatic efficiency and the degradation of acid sensitive glycan moieties.

In addition to RNase B and lactoferrin, HRP was also used as a model glycoprotein. This protein is reported to contain multiple glycosylation sites carrying mainly α1,3-core fucosylated, xylosylated trimannoside [MMXF3 (Manα1-6(Manα1-3)(Xylβ1-2)Manα1-4GlcNAcα1-4(Fucα1-3)GlcNAc)] structures, a glycan typically found in plants and invertebrates [6,7]. The deglycosylation experiments visualized by SDS/PAGE showed that PNGase H^+^ was able to successfully remove the majority of *N*-glycans, whereas PNGase F only deglycosylated negligible amounts of the HRP samples. As shown in Figure 5(C), the 45 kDa protein band of heat-denatured HRP completely shifted to several smaller discrete protein bands with masses between 35 and 40 kDa by PNGase H^+^ treatment, whereas only minute amounts of partially deglycosylated HRP were observed after PNGase F treatment. The partial deglycosylation of a small portion of HRP by PNGase F was also observed by other groups, and is presumably based on the release of glycans lacking α1,3-core fucose [8,33]. According to the UPLC-glycoprofile of HRP samples, PNGase F was not able to release α1,3 core fucosylated *N*-glycans. However, it is noteworthy to say that PNGase F showed a better performance in releasing the xylosylated MMX (Manα1-6(Manα1-3)(Xylβ1-2)Manα1-4GlcNAcα1-4GlcNAc) glycan in comparison with PNGase A. Interestingly, PNGase H^+^ showed a similar performance to PNGase F, and in contrast to PNGase A better performance in the glycan release of MMX (Figure 5B). PNGase H^+^ was therefore shown to be comparable with PNGase A for the release of *N*-glycans containing α1,3-core fucose, and comparable with PNGase F but better than PNGase A for the release of *N*-glycans containing no α1,3-core fucose.

In addition to this observation, an additional minor peak was found in PNGase A treated samples (21.7 min, Figure 5A), which could not be baseline separated from the MMXF3 glycan peak. This minor component is presumably the reason for the second mass peak with the *m*/*z* value of 1473.4 Da [M+Na]^+^ observed in Figure 5(D) (upper part). This *m*/*z* value does not correspond to any HRP glycan structure described so far. MS–MS fragmentation indicated the *N*-glycosidic nature of this compound (Supplementary Figure S3B), indicating that the glycan structure also contains core fucose and xylose, and an unknown modification which adds an additional molecular mass of 142 Da to this glycan in comparison with the MMXF3 glycan. Further exploration of the nature of this unusual glycan mass is currently in progress.

As this minor peak was only present in PNGase A treated HRP samples, it is highly probable that this structure was released by self-deglycosylation from PNGase A itself. Although further study is needed to confirm this, it has indeed been reported that PNGase A possesses core α1,3-fucose and xylose containing *N*-glycans and can be therefore the cause of glycan contamination of treated samples, which is an unavoidable drawback of this enzyme [12]. Despite the uncertainty of the origin of this special peak found in PNGase A treated HRP samples, endogenous glycan interference can be absolutely excluded using PNGase H^+^ and is therefore certainly more preferable than PNGase A. This feature is especially important in probable future applications of PNGases, for instance, treatment of plant foodstuffs to reduce the amount of glycan based allergens.

### Conclusion

We discovered a novel PNGase enzyme from the bacterial strain *T. roseus* DSM 18391. The gene of PNGase H^+^ was successfully cloned and recombinantly expressed in soluble and active form in *E. coli*. The enzyme purification could be optimized to obtain a nearly homogenous enzyme by applying a specifically developed acidic lysis procedure which now enables production in a large scale with low cost. The recombinant PNGase H^+^ demonstrated the enzymatic release of a broad range of *N*-glycans from both glycoproteins and glycopeptides, including structures containing core α1,3-fucose. In addition, PNGase H^+^ is not self-glycosylated and thus has no risk of interfering with sample analysis. It also exhibited very high releasing efficiency of any type of *N*-glycans, which is comparable with both PNGase F and PNGase A. These findings were not reported by any prokaryotic PNGase described so far. This primary report demonstrates that PNGase H^+^ will be a very valuable addition to the currently existing PNGases used for the analysis of *N*-glycans.

## Online data

Supplementary data
