# Supplementary material for: Discovery and characterization of a novel extremely acidic bacterial N-glycanase with combined advantages of PNGase F and A
Source: Biosci Rep. 2014 Nov 14;34(6):e00149. doi: 10.1042/BSR20140148 (PMC4231336; doi:10.1042/BSR20140148)

Supplementary Figure S1. A. 1% Agarose gel image of PCR products. B. DNA sequence and amino acid sequence of recombinant PNGase H^+.

Supplementary Figure S2. MALDI-TOF MS-MS analysis of selected N-glycan fractions derived from HRP.

Supplementary Figure S1:

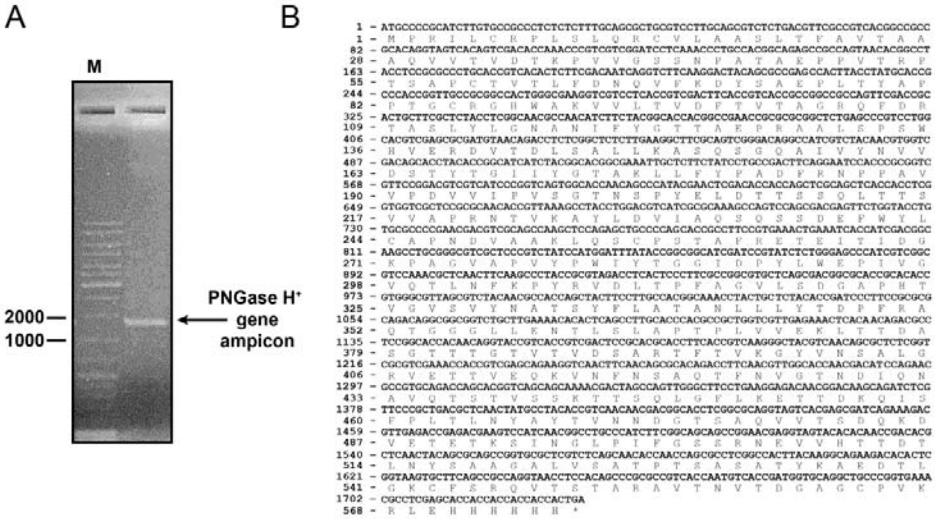

Supplementary Figure S2:

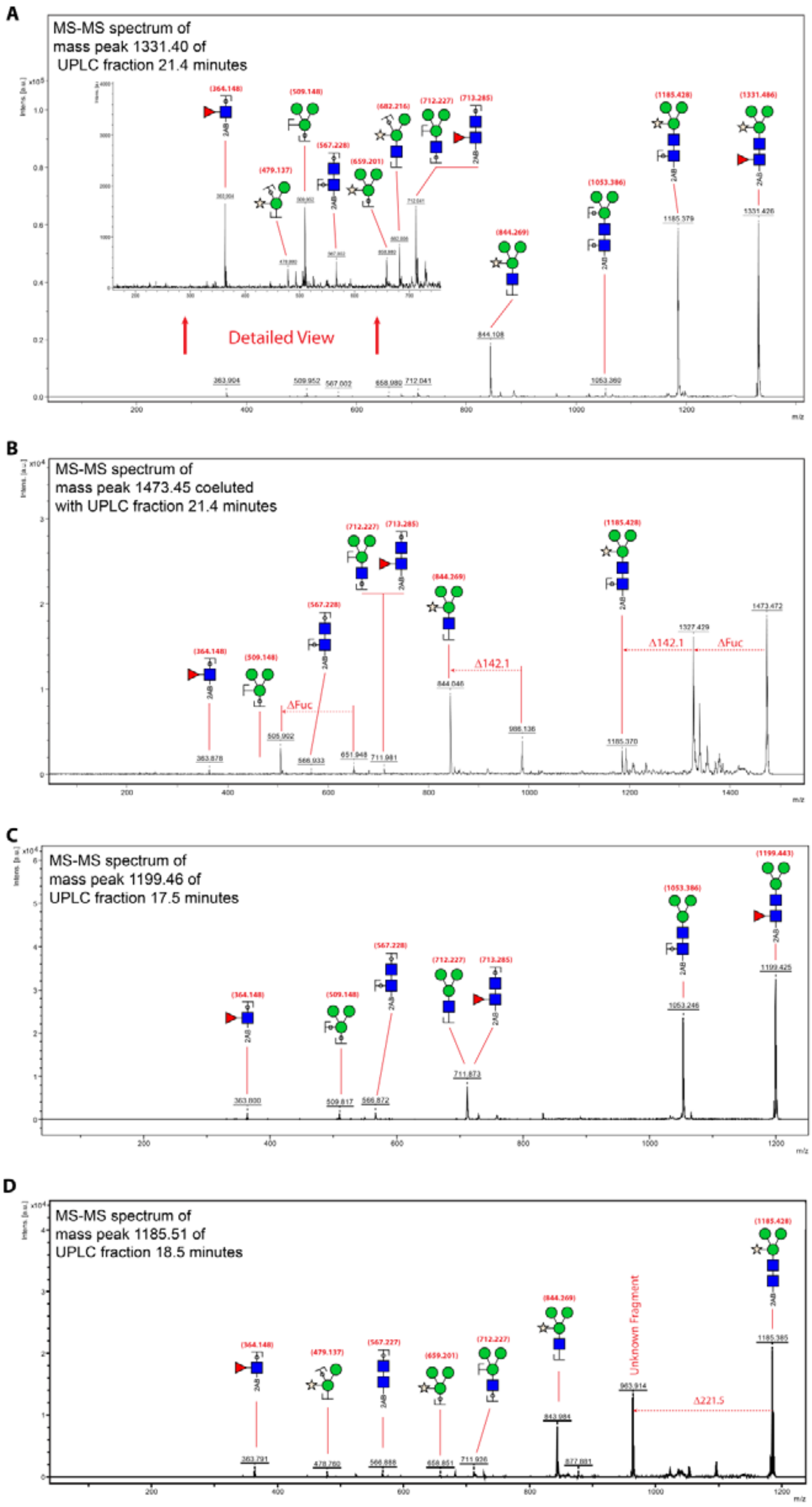

Supplement: Supplementary data [file bsr034e149ntsadd.pdf]
